# Evidence of rustrela virus-associated feline staggering disease in Sweden since the 1970s

**DOI:** 10.1186/s13028-024-00783-5

**Published:** 2024-11-23

**Authors:** Emma Thilén, Dennis Rubbenstroth, Sofia Tengstrand, Florian Pfaff, Jonas Johansson Wensman, Cecilia Ley

**Affiliations:** 1 Department of Animal Biosciences, Faculty of Veterinary Medicine and Animal Science, Swedish University of Agricultural Sciences, P.O. Box 7023, 750 07 Uppsala, Sweden; 2 Institute of Diagnostic Virology, Friedrich-Loeffler-Institut, Südufer 10, 17493 Greifswald – Insel Riems, Greifswald, Germany; 3 Department of Microbiology, Swedish Veterinary Agency, Travvägen 20, 751 89 Uppsala, Sweden

**Keywords:** Borna disease virus, Cat, CNS, Feline ataxia, Immunohistochemistry, Meningoencephalomyelitis, RT-qPCR, RusV

## Abstract

**Background:**

Staggering disease (SD) is a severe neurological disease that has been regularly reported in Swedish cats since the beginning of the 1970s. The aetiology of SD has been debated, but novel rustrela virus (RusV) was recently suggested as the causative agent in Swedish cases dating from 2017 onwards. However, whether RusV was associated with earlier cases of feline SD in Sweden remained unknown. Further, presence of RusV in extraneural tissues of RusV-infected cats and viral transmission routes for RusV are still unknown. Therefore, we investigated the presence of RusV in nervous tissue of historical cases of plausible feline SD in Sweden, dating back to the 1970s, as well as the presence of RusV in selected extraneural tissues.

Formalin-fixed, paraffin-embedded brain and spinal cord from 14 encephalitic cats matching the criteria for SD based on clinical and pathological records, and five non-encephalitic control cats were screened for the presence of RusV antigen and RNA using immunohistochemistry (IHC) and reverse transcription-quantitative polymerase chain reaction (RT-qPCR), respectively. Extraneural presence of RusV antigen was investigated by IHC in four known RusV-positive cats. Morphologic changes were evaluated using light microscopy. In addition, the 14 encephalitic cats were tested for Borna disease virus 1 (BoDV-1) RNA by RT-qPCR.

**Results:**

Morphologic findings compatible with SD were confirmed in 13 of 14 encephalitic cats. All 13 cats were RusV-positive by IHC and 12 of them also by RT-qPCR. One encephalitic cat, morphologically and clinically untypical of SD, as well as all control cats tested negative for RusV RNA and showed either negative or uncertain RusV immunolabeling. There was no firm evidence of extraneural presence of RusV. All encephalitic cats were negative for BoDV-1.

**Conclusions:**

We show that RusV has infected cats in Sweden as far back as the 1970s, whereas BoDV-1 was not detected in any of the investigated cats. This further strengthens RusV as the causative agent of feline SD. Our findings suggest that RusV is strongly neurotropic in cats and that the cat may represent a dead-end host. Further investigations into the pathogenesis of RusV-associated meningoencephalomyelitis in cats are warranted, including disease transmission, pathophysiologic responses and mechanisms of neuronal dysfunction.

**Supplementary Information:**

The online version contains supplementary material available at 10.1186/s13028-024-00783-5.

## Background

Staggering disease (SD) in cats is a severe neurological disease characterized by a staggering gait and hind leg ataxia [1]. Typical microscopic findings are lymphohistiocytic inflammation of the brain, spinal cord and meninges, consistent with non-suppurative meningoencephalomyelitis [1–5]. In Sweden, feline SD was first described in the early 1970s [4]. Since then, the disease has regularly been prevalent in the region of Lake Mälaren and nearby areas [2, 6, 7].

Earlier Swedish studies have suggested Borna disease virus 1 (BoDV-1; species *Orthobornavirus bornaense*; family *Bornaviridae*) as a causative agent of feline SD [1, 6–13]. However, in a recent study including cats from Sweden, Germany and Austria, rustrela virus (RusV; species *Rubivirus strelense*; family *Matonaviridae*) was detected in brain and spinal cord in 27 of 29 tested cats matching the criteria of SD, but not in 29 control cats [2]. Notably, BoDV-1 was not detected in any of the individuals included in that study.

RusV was first described in 2020, when it was detected in nervous tissue of zoo animals with encephalitis in northern Germany [14]. Subsequent reports from Germany on RusV-associated encephalitis included further zoo animal species and a free-living European otter (*Lutra lutra*) [15, 16]. Interestingly, RusV has not only been associated with recent cases of encephalitis, but was also demonstrated in brain tissue of lions (*Panthera leo*) succumbing to fatal encephalitis in two German zoological gardens in the 1980s [17], and in cases of feline SD in Austria from the 1990s [3]. Thus, RusV could potentially also be the aetiology of historic cases of feline SD in Sweden.

The epidemiology of RusV requires further investigation, but several screening studies in a broad range of rodent species together with phylogeographic patterns suggest yellow-necked field mice (*Apodemus flavicollis*) and wood mice (*Apodemus sylvaticus*) as potential reservoir species, whereas encephalitic individuals of other species, including cats, are likely to serve as dead-end hosts [2, 14–18]. Of the suggested reservoirs, only wood mice have so far been shown to harbour the virus in Sweden [2]. Due to its broad spectrum of affected host species, a possible zoonotic potential of RusV cannot be excluded.

Although SD has been occurring in Sweden for several decades, investigations of RusV in Swedish cats have so far been restricted to cases of SD dating from 2017 onwards [2]. In this retrospective study, we aimed to investigate RusV as a potential causative agent of SD in Swedish cats dating back as far as the 1970s, when the first cases in Sweden were recorded [4]. In an attempt to investigate potential transmission routes and/or evidence of strict virus neurotropism, we also investigated the extraneural tissue distribution of RusV in a small number of selected cats, previously diagnosed with RusV-associated meningoencephalomyelitis. Our hypotheses were that RusV is (1) a strongly neurotropic virus in cats and (2) the long-standing leading cause of SD in cats in Sweden.

## Methods

### Compilation of cases and sample selection

The total number of cats examined postmortem at the pathology unit at the Swedish University of Agricultural Sciences (SLU) during 1970–2019 was compiled from archived records. Potential cases of SD were identified based on a postmortem diagnosis of SD, feline ataxia, or a diagnosis of non-purulent (i.e., non-suppurative) meningitis, encephalitis and/or myelitis, and different combinations thereof. Cases diagnosed with SD, feline ataxia and non-suppurative meningoencephalomyelitis were summarized and considered as plausible SD. Cases with other morphological types of inflammation (such as suppurative, fibrinous, and granulomatous) were excluded. Furthermore, cases were excluded if another aetiology (such as *Toxoplasma gondii* or feline infectious peritonitis virus) had been determined or was suggested in the pathology report.

From the cases with plausible SD, three cases from each decade between 1970 and 2016 were randomly selected for confirmation of morphological diagnosis of non-suppurative meningoencephalomyelitis and further investigation of presence of RusV and BoDV-1. Since tissue blocks were available for only two cases from the 1970s, a total number of 14 cases was selected. Cases diagnosed with SD, feline ataxia and non-suppurative meningoencephalomyelitis from 2017 to 2019 were not included in the RusV investigation, as these cases were described previously [2]. Five non-encephalitic control cases, one from each decade from the 1970s to 2010s, were selected from cases lacking a diagnosis of central nervous system inflammatory disease, and for which brain and/or spinal cord tissue had been sampled for histology as part of the postmortem examination. For both plausible SD cases and control cases, cats less than one year were excluded, since feline SD typically presents in adult cats [1, 2, 7]. From each selected case, samples from the cerebrum, cerebellum, brain stem and/or spinal cord were chosen for histology and investigation for presence of RusV.

For the investigation of RusV antigen in extraneural tissues, four known RusV-infected cats, originating from Uppsala County, and included in a previous study were selected, based on tissue availability [2]. Three of these cases originated from 2017 and one from 2019. Lung, liver and kidney were investigated in 4 cats, gastrointestinal tract in 3 cats, spleen and pancreas in 2 cats, and a mesenteric lymph node in 1 cat.

### Histologic examination

Formalin-fixed, paraffin-embedded (FFPE) tissue was sectioned at 4 µm and stained with haematoxylin and eosin. Sections from the brain and spinal cord were evaluated for type and severity of inflammatory perivascular infiltrates. Inflammation was graded as none, mild, moderate, or severe based on a previous protocol [2] (Additional file 1), taking the neuroanatomical localisation into consideration (i.e., cerebrum, cerebellum, brain stem and spinal cord). The meninges in each location were separately evaluated for presence of inflammation.

### Immunohistochemistry for RusV

Approximately 4 µm thick sections were heated to 60 °C for 15 min, before being deparaffinised and rehydrated. For antigen retrieval, a hot water bath (110 °C for 15 min) with a sodium-citrate buffer (pH 6.0) was used. Endogenous peroxidase was blocked with hydrogen peroxide for 5 min in room temperature, and non-specific binding was blocked with normal goat serum (X0907, Dako Denmark ApS, Glostrup, Denmark) for 30 min in room temperature. The sections were incubated with a mouse monoclonal antibody targeting the capsid protein of RusV (#22/2H11B1, kindly provided by Andrea Aebischer, Friedrich-Loeffler-Institut, Griefswald, Germany; hybridoma supernatant diluted 1:20) [2] for 17 h at 4 °C, followed by incubation with a horse radish peroxidase-labelled anti-mouse antibody (Dako EnVision + System, HRP labelled polymer, K4001, Dako North America Inc.) for 30 min in room temperature. Immunolabeling was demonstrated using 3, 3'-diaminobenzidine (Dako Liquid DAB + Substrate Chromogen system, K3468, Agilent Technologies Denmark ApS). The sections were rinsed in tap water, and then counterstained with haematoxylin for one minute. Sections from a known RusV-positive and a known RusV-negative cat [2] were included in all experiments as to ensure accuracy and consistency in immunolabeling results. In a separate experiment, one section from each case with positive RusV immunolabeling was treated with phosphate-buffered saline instead of the primary antibody to ensure that there was no non-specific binding of the secondary antibody. Grading of immunolabeling was based on a previously published protocol [2] (Table 1).

**Table 1:** Criteria for grading of immunolabeling for rustrela virus in brain and spinal cord

Grade	Criteria
0	No immunolabeling or minor uncertain immunolabeling
1	Weak or distinct immunolabeling in few neurons and/or glial cells
2	Weak immunolabeling in multiple neurons and/or glial cells
3	Distinct immunolabeling in multiple neurons and/or glial cells

Modified from Matiasek et al. [2]

### Detection of RusV and BoDV-1 RNA by RT-qPCR

FFPE material from the cerebrum, cerebellum and brain stem from all 14 encephalitic cats and all five non-encephalitic control cats were investigated for RusV RNA. In some tissue blocks, spinal cord was embedded together with brain samples and thus included as part of the analysis.

RNA extraction from FFPE scrolls was performed using the RNeasy FFPE Kit (Qiagen, Hilden, Germany) with Deparaffinization Solution (Qiagen) according to the manufacturer´s instructions. Detection of RusV RNA was performed by RT-qPCR assay panRusV-2a, which is a modification of the previously published assay panRusV-2 [2] with an altered TaqMan probe (RusV_257_P; 5'-[FAM]TGAGCGACCACCCAGCACTCCA[BHQ1]-3'). Primers and PCR conditions were identical to those described previously [2].

Samples from all 14 encephalitic cats were additionally tested for the presence of BoDV-1 RNA by RT-qPCR assays BoDV-1 Mix-1 and Mix-6 as described previously [19, 20].

### Sequencing of RusV and phylogenetic analysis

Generation of partial RusV p150-encoding sequences was attempted for all RusV-positive animals by Sanger sequencing of four overlapping conventional RT-PCR amplicons of 142 to 191 base pairs lengths, as previously described [17]. Each amplicon was sequenced in both directions. Assembly of raw sequences trimmed for quality and primer sequences resulted in a sequence of 409 nucleotides (nt) length (representing genome positions 100 to 508 of RusV reference genome MN552442.2). The final sequences were deposited in GenBank under accession numbers PP910111 to PP910113.

Phylogenetic analysis of these sequences was performed together with all previously published RusV sequences for which the respective sequence stretch of 409 nt length was available in GenBank (n = 64). The sequences were aligned using MUSCLE 3.8.425 (available in Geneious Prime 2021.0.1, Auckland, New Zealand). Subsequently, a Neighbor-Joining phylogenetic tree was calculated using MEGA (version 11.0.13; Tamura-Nei method with a gamma distribution; shape parameter = 1; 1,000 bootstrap replicates) [21].

## Results

### Numbers of cats with non-suppurative inflammation of the brain, spinal cord and/or meninges

**Fig. 1:**
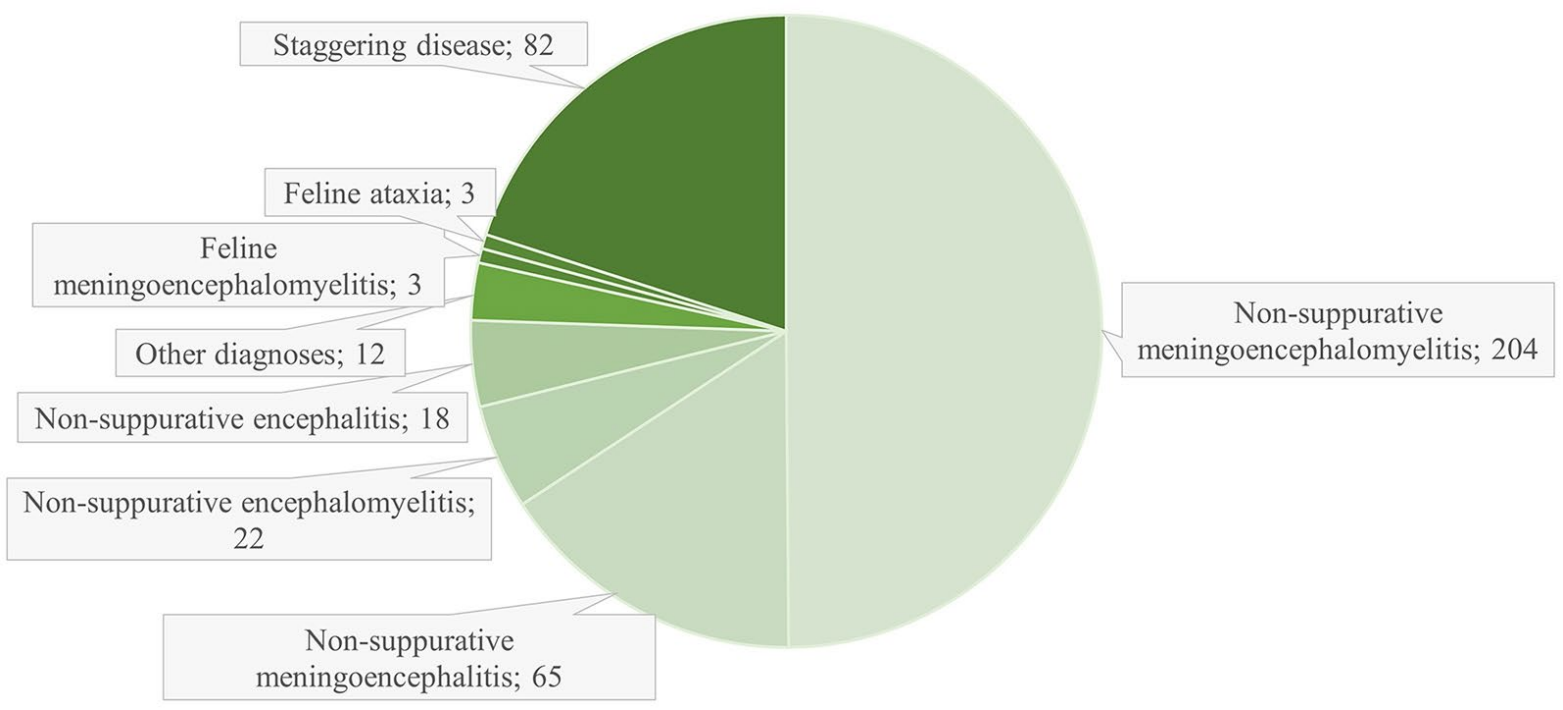
Numbers of cats with suggested or potential staggering disease according to postmortem reports (1970–2019). The category “Other diagnoses” includes the diagnoses: non-suppurative meningitis (6 cats), non-suppurative spinal meningitis (1 cat), non-suppurative meningomyelitis (3 cats), non-suppurative myelitis (1 cat), and lymphoplasmacytic meningoencephalomyelitis (1 cat)

**Fig. 2:**
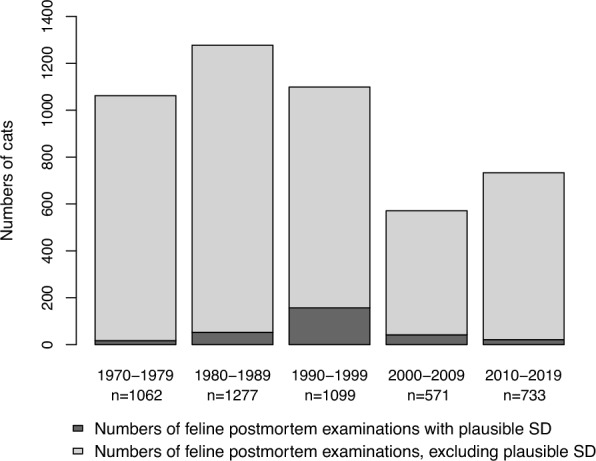
Numbers of cats per decade with plausible staggering disease (SD). n = total number of feline postmortem examinations

In total, 4,742 cats were examined postmortem at SLU from 1970 to 2019, 409 (8.6%) of which had a diagnosis compatible with SD, feline ataxia, or non-suppurative meningitis, encephalitis and/or myelitis or different combinations thereof (Fig. 1). The highest number of cases with plausible SD (i.e., cases with a diagnosis of either SD, feline ataxia or non-suppurative meningoencephalomyelitis) occurred in the 1990s when 157 cases were recorded (corresponding to 14.3% of total number of feline postmortem cases) (Fig. 2). A peak in plausible SD cases occurred in 1993, when 47 cases were recorded (data not shown). The lowest number of cats with plausible SD was recorded in the 1970s with 17 cases (corresponding to 1.6% of total number of feline postmortem cases). For the remaining decades, plausible SD accounted for 52 cases (4.1%) in the 1980s, 42 cases (7.4%) in the 2000s, and 21 cases (2.9%) in the 2010s.

### Demographic data

For the 14 plausible SD cases investigated for RusV, ages ranged from 2 to 10 years (mean age 5.4 years). There were two intact females, two intact males, six neutered females and four neutered males. Seven were Domestic shorthair, three European shorthair and one Burmese (missing information for three cats). Based on the area code given in the postmortem submission form, 11 cats were from Uppsala County and two from the adjacent Stockholm County (missing information for one cat). The most common clinical signs were hind limb ataxia, behavioural changes, a staggering gait, decreased postural reflexes, pain from the lumbar and sacral region and inappetence. Demographic data and details on clinical signs and duration of disease are provided in Additional file 2.

For the five non-encephalitic cats included as controls, ages ranged from 3 to 18 years (mean age 9.4 years). There were three intact males and two neutered males. Two cats were Domestic shorthair and two European shorthair (missing information for one cat). Three cats were from Uppsala County (missing information for two cats). Signalment and postmortem diagnosis for control cats are provided in Additional file 3.

### Histological findings

**Fig. 3:**
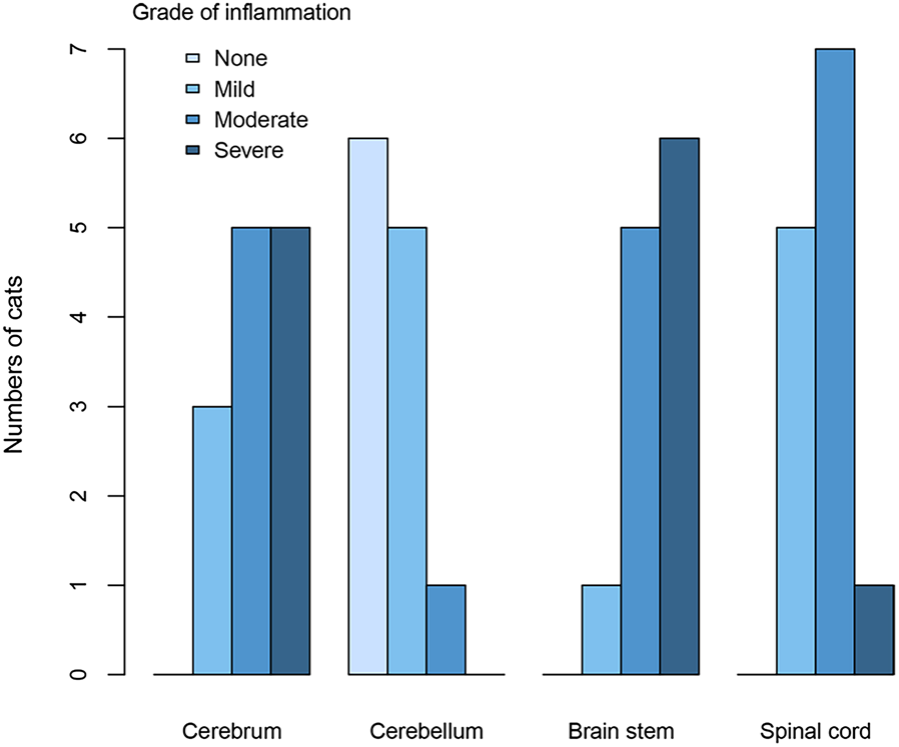
Grade of inflammation per neuroanatomical location in cats with non-suppurative meningoencephalomyelitis

**Fig. 4:**
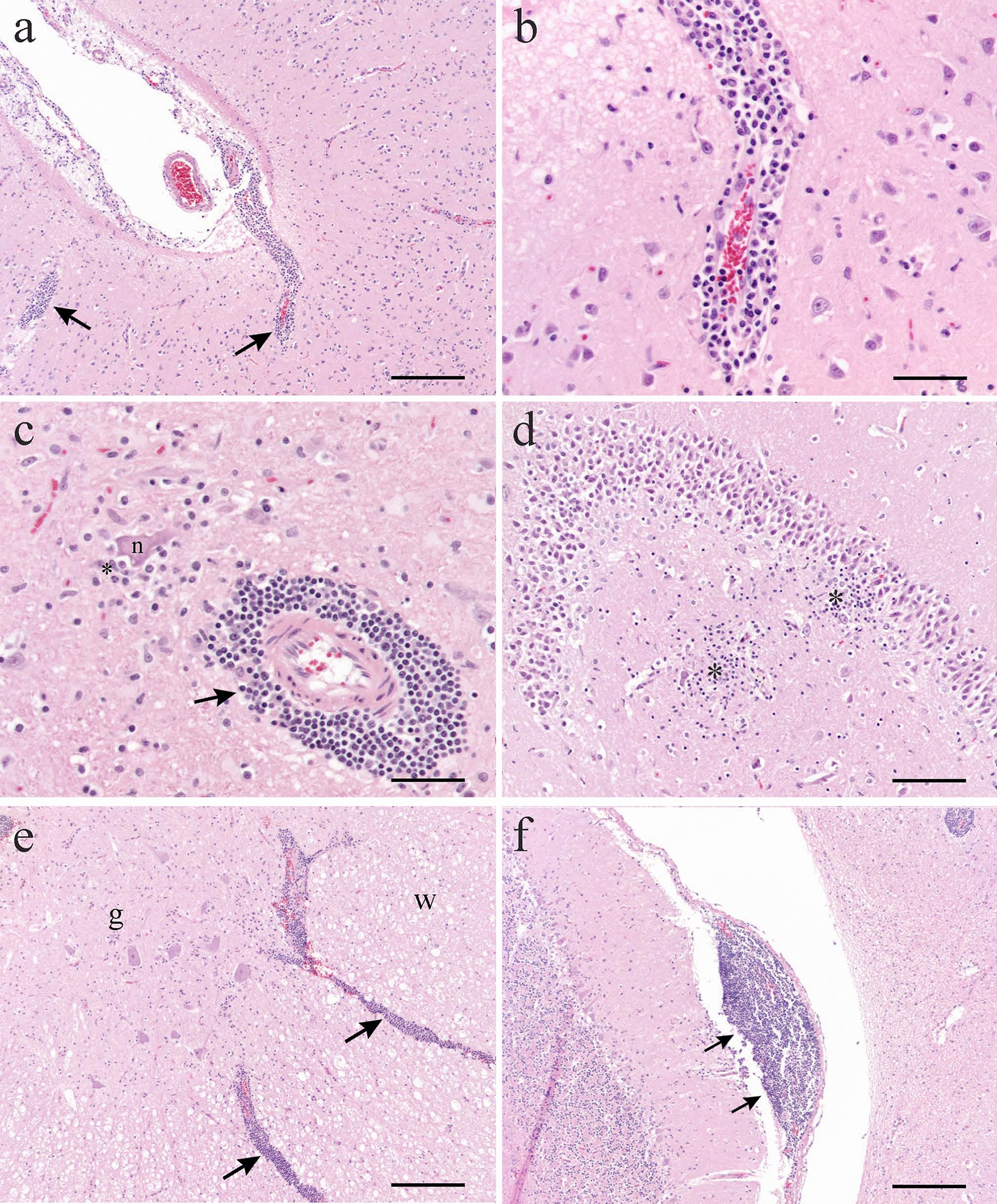
Photomicrographs of brain and spinal cord sections from cats with non-suppurative meningoencephalomyelitis and plausible staggering disease. **a)** Inflammatory infiltrates (arrows) located in the cerebral grey matter and meninges in cat No. 1978a. **b)** Higher magnification of perivascular inflammatory infiltrate in a) showing a mixed population of lymphocytes, histiocytes and plasma cells. **c)** Degenerative neuron (n) surrounded by glial cells (*) and perivascular inflammatory infiltrate (arrow) in brain stem from cat No. 1993. **d)** Glial proliferations in the hippocampus (*) in cat No. 2015. **e)** Perivascular infiltrates following vessels in grey (g) and white (w) matter in spinal cord from the same cat as depicted in a). **f)** Large inflammatory infiltrate (arrows) located in the meninges between the cerebellum and brain stem with additional perivascular inflammatory infiltrate present in brain stem (upper right corner) in cat No. 1978b. In **a**), **e**) and **f**) bar = 200 µm, in **b**) and **c**) bar = 50 µm and in **d**) bar = 100 µm. Haematoxylin and eosin stain

Non-suppurative meningoencephalomyelitis was confirmed in 13 of the 14 cats with plausible SD (Table 2 and Fig. 3). Perivascular inflammatory infiltrates were dominated by lymphocytes and histiocytic cells, with occasionally smaller numbers of plasma cells and a few macrophages with intracytoplasmic lipid-like material. Perivascular inflammatory cells were predominantly located in the grey matter of the cerebrum, brain stem and spinal cord, and in the meninges. Perivascular inflammatory infiltrates were most pronounced in the cerebrum and brain stem (Fig. 4a-c), and sometimes infiltrates extended from perivascular locations into the adjacent neuropil. Glial proliferation was commonly detected (Fig. 4d), whereas neuronal degeneration with satellitosis was less common (Fig. 4c). When present in sections, the thalamic region often showed marked perivascular inflammatory infiltrates, whereas infiltrates in the hippocampus were typically milder. The spinal cord most often showed moderate or mild perivascular inflammatory infiltrates (Table 2 and Fig. 3), and these infiltrates sometimes followed perivascular spaces extending between grey and white matter regions (Fig. 4e). The cerebellum often showed no or mild perivascular inflammatory infiltrates (Table 2 and Fig. 3). Meningeal inflammatory infiltrates comprised both diffusely scattered cells and larger perivascular aggregates, the latter often, but not exclusively, associated with the meninges of the cerebellum (Fig. 4f). When seen in the cerebellar region, cellular aggregates were predominately located in cerebellar sulci.

**Table 2:** Grades of inflammation and immunolabeling for rustrela virus (RusV) in cats with non-suppurative meningoencephalomyelitis

Case No.	Cerebrum		Cerebellum		Brain stem		Spinal cord	
	Infl	RusV IHC	Infl	RusV IHC	Infl	RusV IHC	Infl	RusV IHC
1978a	Severe	3	Mild	2	Severe	2	Moderate	2
1978b	Moderate	3	None	2	Moderate	2	Moderate	2
1980	Severe	0	None	2	Severe	2	Moderate	0
1983	Moderate	2	None	3	Mild	3	Mild	2
1984	Mild	2	n/a	n/a	n/a	n/a	Mild	2
1990	Moderate	0	Mild	3	Moderate	1	Mild	0
1993	Severe	1	Mild	3	Severe	1	Moderate	0
1996	Mild	3	None	3	Moderate	3	Mild	3
2003	Moderate	3	None	2	Moderate	2	Moderate	3
2004	Mild	1	Mild	1	Severe	2	Moderate	0
2009	Severe	0	Mild	1	Severe	1	Moderate	1
2015	Moderate	3	None	3	Moderate	3	Mild	3
2016	Severe	3	Moderate	3	Severe	3	Severe	3

*IHC* immunohistochemistry, *Infl*. inflammation, *n/a* not applicable (missing sample)

One of the 14 encephalitic cats (No. 2014) showed a neutrophil-rich inflammatory reaction atypical of SD and had hence been erroneously diagnosed as a non-suppurative meningoencephalomyelitis at the initial postmortem examination. Cat No. 2014 was therefore excluded from grading of inflammation.

In all control cats, sections from the cerebrum, cerebellum and brain stem were examined. In two control cats where tissue from spinal cord was available this was included as well. Infiltration of small numbers of inflammatory cells, scattered and/or perivascularly distributed in the meninges and/or the neural tissue, were observed in three cats: one cat diagnosed with acute myelomalacia and ligament/muscle ruptures with haemorrhages surrounding thoracic vertebrae, one cat diagnosed with discospondylitis, and one cat diagnosed with a meningioma. In the two remaining control cats, evidence of inflammation was not present.

### Detection of RusV capsid protein in brain and spinal cord

**Fig. 5:**
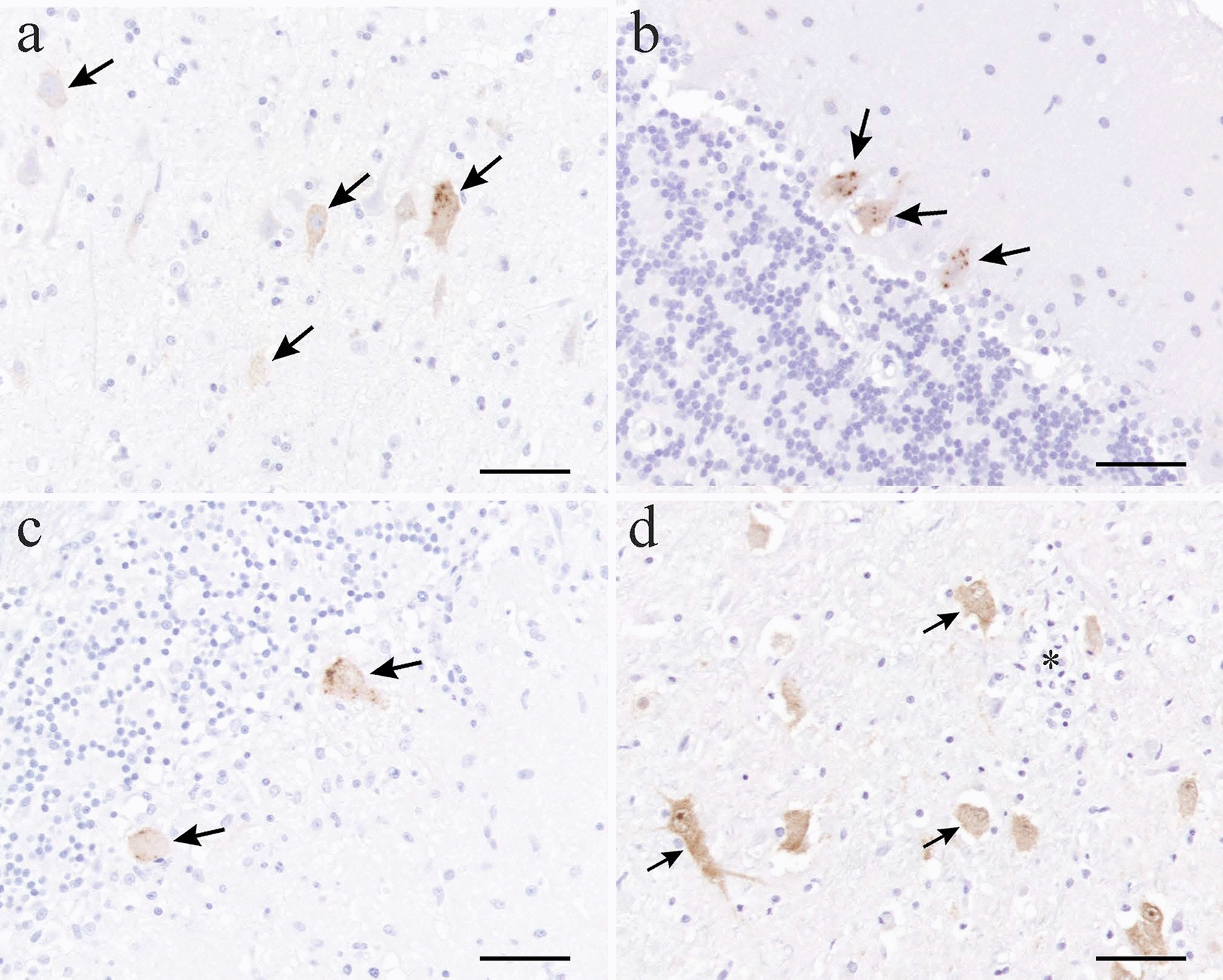
Photomicrographs of brain sections immunolabeled for rustrela virus (RusV) from cats with non-suppurative meningoencephalomyelitis. Positive immunolabeling (grade 3) for RusV (arrows) in cerebral neurons in cat No. 1978b (**a**), in Purkinje cells of the cerebellum in cat No. 1983 (**b**) and cat No. 1993 (**c**), and in neurons in the thalamus in cat No. 2003 (**d**), glial nodule indicated by *. Bar = 50 µm

Immunolabeling for RusV was detected in two or more locations in all 13 cats with non-suppurative meningoencephalomyelitis (Table 2 and Additional file 4). Immunolabeling was observed in the cytoplasm of neurons of the cerebral cortex, hippocampus, brain stem and spinal cord, and in Purkinje cells of the cerebellum (Fig. 5), and typically immunolabeled cells appeared morphologically viable. Immunolabeling was occasionally observed in glial cells, and minor immunolabeling was sometimes detected in the neuropil and white matter. Immunolabeling was less common in the spinal cord compared to the other investigated locations (Table 2 and Additional file 4). No immunolabeling was detected in cat No. 2014 showing neutrophil-rich meningoencephalomyelitis and in four of the five non-encephalitic control cats. One control case exhibited a sparse amount of cytoplasmic brown granules in a few Purkinje cells (immunolabeling graded 0).

### Detection of RusV capsid protein in extraneural tissue

Distinct immunolabeling for RusV was observed in a single cell in the mucosa of the small intestine of cat No. 2017a, whereas all other investigated tissues were negative (Additional file 5).

### Detection of RusV- and BoDV-1-specific RNA by RT-qPCR

RusV-specific RNA was detected by RT-qPCR in the brain of 12 of the 13 cats with a non-suppurative meningoencephalomyelitis, and Cq values ranged from 25.4 to 37.3 (Additional file 4). No RusV-specific RNA could be detected in the brains of the five control cats and of cat No. 2014, which showed a neutrophil-rich meningoencephalomyelitis (Additional file 4). BoDV-1-specific RNA was not detected in the samples from any of the 14 cats with plausible SD (data not shown).

### Sequencing of RusV

**Fig. 6:**
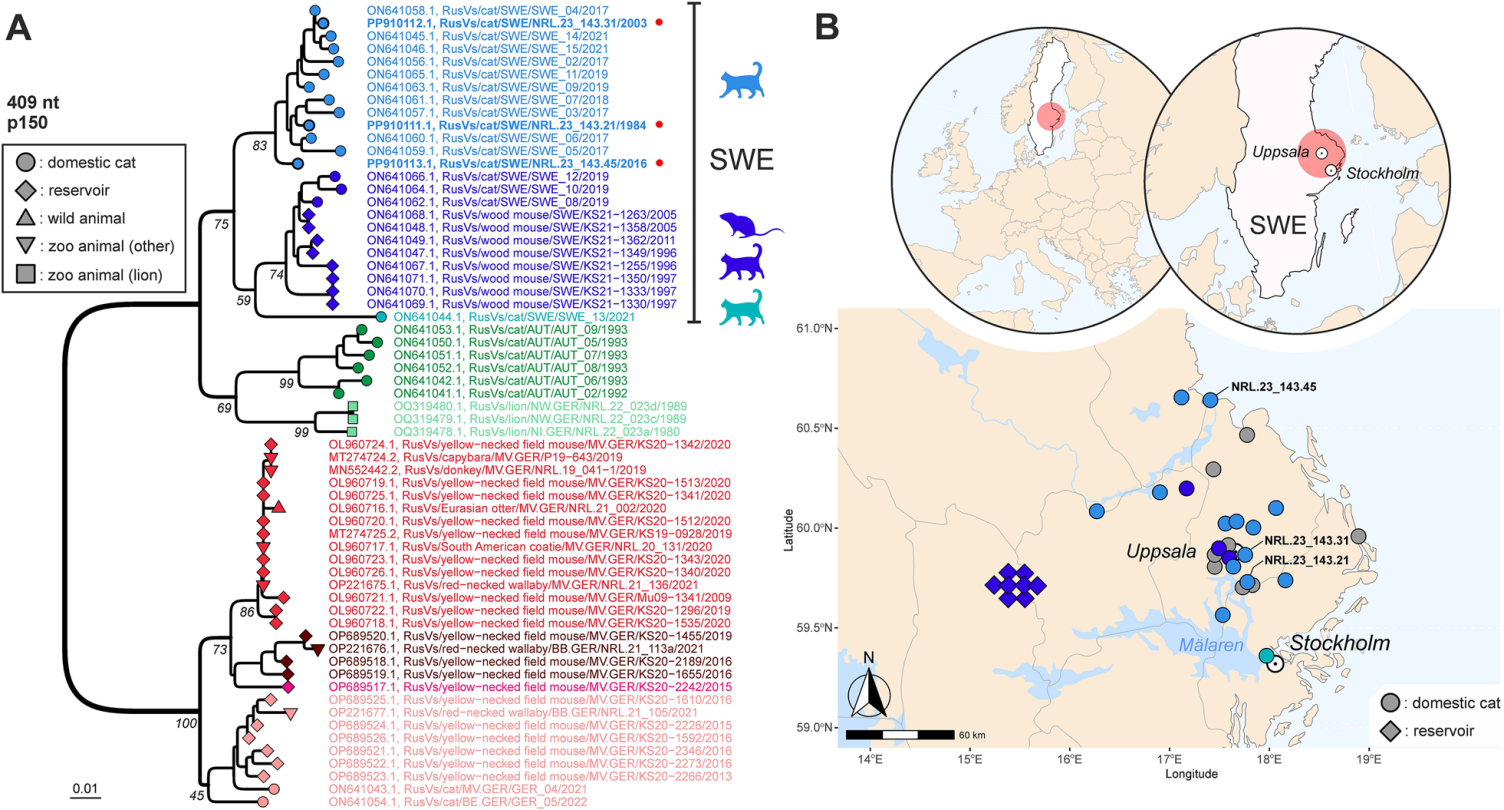
Phylogenetic analysis and spatial distribution of rustrela virus (RusV) infections in Europe. **A** Phylogenetic analysis of rustrela virus (RusV) infections in Europe. Neighbor-Joining phylogenetic tree of partial RusV p150 sequences (409 nt; MEGA version 11.0.13; TN93 + G + I; 1,000 bootstrap replicates). Only bootstrap values at major branches are shown in the phylogenetic tree. RusV sequence names are shown in the format “host/ISO 1366 code of location (federal state.country)/animal ID/year”. Sequences generated during this study are depicted in bold and marked with a red dot. **B** Mapping of the geographic origin of RusV-positive animals in Sweden. Colours represent the phylogenetic clades of the sequences. Grey circles represent RusV-positive cats investigated in this study without available sequence, whereas the three cats with available sequences are indicated by NRL code. White circles with central dot mark the location of Uppsala and Stockholm. The respective hosts are indicated by circles (domestic cats), squares (lions), triangle (wild animals), upside-down triangles (zoo animals) or diamonds (*Apodemus* spp.). *AUT* Austria, *GER* Germany, *SWE* Sweden, *BB* Brandenburg, *BE* Berlin, *MV* Mecklenburg-Western Pomerania, *NI* Lower Saxony, *NW* North Rhine-Westphalia. Made with Natural Earth

Sequencing of a 409 bp stretch of the RusV genome was attempted for all RT-qPCR-positive cats by Sanger sequencing of four short overlapping RT-PCR amplicons. The intended 409 bp sequence was entirely determined only for the three cases with the lowest RT-qPCR Cq values (cats No. 1984, 2003 and 2016). Phylogenetically, all three sequences belonged to the same clade composed of RusV sequences from cats mainly from Uppsala province, but also individual animals from the provinces Stockholm, Dalarna and Gävleborg (Fig. 6). Shorter sequence fragments were yielded for three additional cats. Preliminary analysis suggested these sequences to likewise belong to the Swedish cluster of RusV (data not shown). No RusV sequences were obtained for the remaining seven RusV-positive animals.

## Discussion

Cases of SD in cats have been observed in Sweden throughout the last five decades [1, 2, 4, 6–12, 22]. Recently, SD was demonstrated to be associated with RusV infection based on cases diagnosed in Sweden from 2017 onwards [2]. However, whether RusV was also associated with feline SD occurring in Sweden prior to 2017 was not investigated. We therefore searched for RusV antigen and RNA in archived brain and spinal cord samples from historic cases of cats with plausible SD and non-suppurative meningoencephalomyelitis, dating back as far as the 1970s, when SD in cats was first reported in the literature [4].

We detected RusV antigen by IHC in all 13 selected cats with non-suppurative meningoencephalomyelitis and clinical signs consistent with SD. RusV RNA could be detected by RT-qPCR in FFPE brain tissue from 12 of these cases. In contrast, only one of the five non-encephalitic control cats showed minor uncertain immunolabeling, but no RusV RNA was detected. Furthermore, RusV antigen and RNA was not detected in the cat suffering from a neutrophil-rich meningoencephalitis, and in this cat neither morphologic findings nor clinical signs were typical of SD. BoDV-1 RNA was not detected in the brains of the cats with signs of SD. In agreement with previous studies [2, 3], these results further corroborate RusV to be the causative agent of feline SD, although Henle/Koch’s postulates yet need to be fulfilled.

Overall, most samples showed high RT-qPCR Cq values, representing low amounts of viral RNA, which may be attributed to fixation and long-term storage of the FFPE samples resulting in RNA degradation [23]. Samples with strong immunolabeling (grade 3) on IHC generally presented lower Cq values compared to samples with weaker immunolabeling. One IHC-positive cat showed a negative RT-qPCR-result. This case showed immunolabeling in multiple cerebellar Purkinje cells and scattered neurons of the brain stem, suggesting that this animal nonetheless was RusV-infected. The sparse brown cytoplasmic granules in a few Purkinje cells of the cerebellum of one RT-qPCR-negative non-encephalitic control animal may rather suggest an artefactual staining. However, the possibility of RusV infections in cats without development of meningoencephalomyelitis or clinical signs cannot be excluded. Further research is required to investigate if cats that do not show clinical signs of SD can carry RusV, and if so, what the predisposing factors are for development of clinical disease.

In addition, we performed a limited investigation of the presence of RusV in extraneural tissues from four cats with known RusV-associated meningoencephalomyelitis [2], which had undergone postmortem examination in 2017 or 2019. In accordance with the absence of RusV antigen in extraneural tissues in RusV-associated meningoencephalitis in lions [17] and red-necked wallabies [15] in zoos in Germany, immunolabeling was only observed in a single cell in the mucosa of the small intestine in one investigated cat in the present study. The lack of immunolabeling in extraneural tissues may indicate that RusV is a strictly neurotropic virus in cats, and that cats represent a dead-end host for RusV. However, extended evaluations of presence of RusV in additional extraneural tissues are warranted to further support this theory.

In agreement with previously described morphologic characteristics of SD, the grey matter of the cerebral cortex, brain stem and spinal cord were predominately affected in RusV-positive animals [1, 2, 4, 5], with meningitis being prominent in the cerebellar region, but also seen in cerebral and spinal regions [1, 4, 5]. Overall, perivascular inflammatory infiltrates were most often graded as moderate, with severe inflammatory changes most commonly detected in the brain stem and the cerebrum. Variable, but generally milder, perivascular inflammatory infiltrates were detected in the spinal cord, whereas they were rather scarce in the cerebellum. The reason for this distributional pattern is unclear, but is in accordance with previously described [1, 2, 4, 5, 10]. Interestingly, sections from the spinal cord more often showed a negative result for RusV with IHC compared to sections from the brain, which may indicate that RusV has a higher tropism for brain neurons compared to spinal cord neurons. Furthermore, neurons showing immunolabeling for RusV tended to be morphologically intact. This could indicate that clinical signs in RusV-associated meningoencephalomyelitis are related to either inflammatory-induced neuronal injury or that RusV primarily cause deficits in neuronal functions rather than cell death.

Our screening of the diagnostic records of the SLU pathology unit identified a total of 409 cats with potential SD based on morphologic diagnoses established at postmortem examination, representing approximately 9% of all cats examined postmortem at SLU during 1970–2019. The pathology unit at SLU is currently situated in Uppsala County, after being relocated from Stockholm County in 1976. Lake Mälaren region, where SD is regularly encountered, stretches across both of these counties. Since the pathology unit at SLU mainly receives cases with this regional origin, it was not unexpected that the case load of feline postmortem examinations comprised a relatively high percentage of cats with plausible SD. Indeed, all cases with known postal codes investigated for presence of RusV in the present study were from Uppsala and Stockholm County. The number and proportion of potential SD cases examined at SLU was markedly elevated in the 1990s. This may, at least partly, be related to increased awareness for the disease and active recruitment of cats with suspected SD for postmortem examination due to research projects on feline SD performed at SLU in the beginning of the 1990s [22]. The considerable number of cases consistent with the characteristics of feline SD in postmortem examinations at SLU further shows that feline SD is still of major concern for feline health in this geographic part of Sweden. Whether RusV-associated meningoencephalomyelitis also occurs in other regions in Sweden and/or in other animal species, similar to reports from Germany [14–17], is currently unknown and warrants further investigation. Considering that the cats with non-suppurative meningoencephalomyelitis investigated for RusV in the present study were randomly selected, and that all were positive for RusV, we suspect that RusV-associated SD has been a common feline neurological disease in Sweden ever since SD was first reported in the 1970s [4].

BoDV-1 has been suggested as a causative agent of feline SD [6, 8–12, 22, 24]. However, neither BoDV-1 RNA nor antigen could be detected in any of the cases included in previous studies examining cats with SD from Sweden, Germany and Austria [2, 3]. Likewise, we were not able to detect BoDV-1 RNA in any of the cases included in the current study, which calls for a re-evaluation of previous research regarding the aetiology of feline SD.

This study has some limitations. The compilation of cases with potential SD at SLU between 1970 and 2019 was largely based on the postmortem diagnosis and information on clinical histories was not recorded in the vast majority of cases. In addition, the morphologic diagnosis was verified only for those 14 cases selected for the RusV screening. Thus, it cannot be ruled out that some cases of non-suppurative inflammation of the brain and/or spinal cord would be related to other diseases than SD. A recent study in cats with non-suppurative encephalomyelitis from Austria detected RusV in a minority of investigated animals [3], suggesting that other pathogens may cause disease morphologically similar to RusV-associated feline SD. However, in the Austrian study [3], selection criteria for the investigated cases based on typical clinical signs of SD were less stringent, why finding only few cases of RusV-positive animals was not unexpected. Further, due to the retrospective nature of the current study, the morphologic evaluation and investigations for presence of RusV antigen and RNA were based on tissue that had been sampled in different locations by different pathologists. Due to the heterogeneous sample collection, it was not possible to examine the exact same anatomic location of the central nervous system in all cats. Thus, the results from grading of inflammation and RusV detection could be influenced by variation in locations. It would be desirable to perform more systematic analyses of immune reactions, cellular changes and viral loads in specific brain and spinal cord regions in future studies of the pathogenesis of feline SD.

## Conclusions

We show that RusV has been infecting cats in Sweden since the 1970s and provide evidence suggestive of RusV being the common and long-standing cause of feline SD. Detection of viral antigens in neurons lacking morphological evidence of degeneration warrants further investigations regarding the pathogenesis and development of clinical signs. Lack of RusV in extraneural tissues may suggest that the virus is strictly located in central nervous tissue of cats and that the cat represents a dead-end host.

## Supplementary Information

Additional file 1: Grading of perivascular inflammation in brain and spinal cord. File format: Microsoft Word. File extension.

Additional file 2: Demographic and clinical data in cats with plausible staggering disease. File format: Microsoft Word. File extension.

Additional file 3: Demographic data and postmortem diagnosis for non-encephalitic control cats. File format: Microsoft Word. File extension.

Additional file 4: Detection of rustrela virus (RusV) in formalin-fixed, paraffin-embedded brain and spinal cord from cats with non-suppurative meningoencephalitis, controls and reference cases File format: Microsoft Word. File extension.

Additional file 5: Immunolabeling for rustrela virus (RusV) in extraneural tissue from four known RusV-positive cats^*a*^. File format: Microsoft Word. File extension.

## Data Availability

The datasets used and/or analysed during the current study are available from the corresponding author on reasonable request. RusV sequences generated during this study have been deposited in GenBank under accession numbers PP910111 to PP910113.

## Abbreviations

BoDV-1: Borna disease virus 1
FFPE: Formalin-fixed, paraffin-embedded
IHC: Immunohistochemistry
RT-qPCR: Reverse transcription quantitative polymerase chain reaction
RusV: Rustrela virus
SD: Staggering disease
SLU: Swedish University of Agricultural Sciences

## Declarations

### Prior publication

Data included in this article have previously been published in an abstract in the SLU archive for student projects (Epsilon, 7 February 2024) and in the proceedings of the 46th Nordic Society of Veterinary Pathology Symposium and Meeting, Oscarsborg, Norway, 12–14 June 2024. Additionally, the study was presented as an oral presentation at the Cutting Edge Pathology Congress, San Lorenzo de El Escorial, Spain, 28–31 August 2024.
