# Supplementary material for: Evidence of rustrela virus-associated feline staggering disease in Sweden since the 1970s
Source: Acta Vet Scand. 2024 Nov 23;66:59. doi: 10.1186/s13028-024-00783-5 (PMC11585236; doi:10.1186/s13028-024-00783-5)
Supplement: Supplementary file 1 — Additional file 1: Grading of perivascular inflammation in brain and spinal cord. File format: Microsoft Word. File extension. [file 13028_2024_783_MOESM1_ESM.docx]

**Additional file 1.** Grading of perivascular inflammation in brain and spinal cord

| Grade | Criteria |
| --- | --- |
| None | No perivascular infiltrates |
| Mild | Few perivascular infiltrates, most of which show 1-2 layers of cells |
| Moderate | Several perivascular infiltrates showing 3-5 layers of cells, allowing occasional larger or smaller infiltrates |
| Severe | Many perivascular infiltrates, with several showing >5 cell layers of cells |

Modified from *Matiasek et al*. [2].
