# Supplementary material for: Evidence of rustrela virus-associated feline staggering disease in Sweden since the 1970s
Source: Acta Vet Scand. 2024 Nov 23;66:59. doi: 10.1186/s13028-024-00783-5 (PMC11585236; doi:10.1186/s13028-024-00783-5)
Supplement: Supplementary file 2 — Additional file 2: Demographic and clinical data in cats with plausible staggering disease. File format: Microsoft Word. File extension. [file 13028_2024_783_MOESM2_ESM.docx]

**Additional file 2.** Demographic and clinical data in cats with plausible staggering disease

| **Duration of  clinical signs** | 5 weeks | 1 year | N.S. | 1.5 months | 3–4 days or more | 1 month or more | About 3 weeks | Behavioural changes for 5 yrs | 4 days | 1 month | 2 months | 2 days | 8-10 months | 4–5 days |
| --- | --- | --- | --- | --- | --- | --- | --- | --- | --- | --- | --- | --- | --- | --- |
| **Main clinical signs** | Inappetence; ataxia, hind extremities; increased affection; increased vocalisation | Progressing paraplegia, hind extremities | Ataxia, hind extremities; reluctance to jump; withdrawn | Ataxia, hind extremities; muscle atrophy, hind extremities; pain in caudal lumbar region; decreased postural reactions; reduced ability to jump; inability to retract claws; increased vocalisation | Ataxia, hind limbs; staggering gate; inappetence; reduced ability to jump; nasal discharge; tachypnea and abdominal breathing | Ataxia, hind limbs; staggering gate; unwilling to climb stairs; quiet vocalisation; urinating and defecating outside litter box | Staggering gate; initial swelling, right hind extremity | Ataxia, hind extremities; behavioural changes; failiure to groom coat; uringating and defecating outside litter box | Ataxia, hind extermities; stiffness, front extremities; neurological deficiets all extremities | Ataxia, hind extremities; kyphosis; stiff gait; fever; pain in lumbar region; inability to retract claws; inappetence: increased vocalisation; absent flexor reflex, hind extremities; absent anal and vulvourethral reflex | Ataxia, hind extremities; muscle atrophy, especially hind extremities: reduced ability to jump, decreased postural reactions; contact seeking; weight loss despite good appetite | Day 1: recumbent, staring gaze and unresponsive to stimuli. Day 2: development of shock with bradycardia, reduced consciousness, tachypnea, reduced menace and palpebral reflexes; dies | Staggering gate; fever; inappetence; coprostasis | Ataxia, hind extremities; staggering gate; reduced postural reactions front legs; lethargy; pain in caudal lumbar region; miosis; normal pain response, all extremities |
| **Age  (yrs)** | 8 | 7 | 3 | 4 | 4 | 5 | 4 | 7 | 10 | 3 | 5 | 3 | 2 | 10 |
| **Sex** | M | F, n | M, n | F, n | F | M | M, n | F | M, n | F, n | F, n | F, n | M, n | F, n |
| **Breed** | EUS | N.S. | EUS | N.S. | EUS | N.S. | DSH | BC | DSH | DSH | DSH | DSH | DSH | DSH |
| **Location of origin** | Skärplinge | N.S. | Uppsala | Uppsala | Björklinge | Uppsala | Vattholma | Väddö | Uppsala | Björklinge | Knivsta | Norrtälje | Tierp | Skutskär |
| **Case**  **No.** | 1978a | 1978b | 1980 | 1983 | 1984 | 1990 | 1993 | 1996 | 2003 | 2004 | 2009 | 2014* | 2015 | 2016 |

* = cat showing neutrophil-rich inflammatory changes atypical of staggering disease, BC: Burmese cat, DSH: Domestic shorthair, EUS: European shorthair, F: female, M: male, n: neutered, N.S.: not stated, yrs: years.
