# Supplementary material for: Evidence of rustrela virus-associated feline staggering disease in Sweden since the 1970s
Source: Acta Vet Scand. 2024 Nov 23;66:59. doi: 10.1186/s13028-024-00783-5 (PMC11585236; doi:10.1186/s13028-024-00783-5)
Supplement: Supplementary file 3 — Additional file 3: Demographic data and postmortem diagnosis for non-encephalitic control cats. File format: Microsoft Word. File extension. [file 13028_2024_783_MOESM3_ESM.docx]

**Additional file 3.** Demographic data and postmortem diagnosis for non-encephalitic control cats

| Case No. | Location of origin | Breed | Sex | Age (years) | Diagnosis |
| --- | --- | --- | --- | --- | --- |
| C1977 | Uppsala | European shorthair | M | 3 | Acute myelomalacia; ligament/muscle ruptures with haemorrhages surrounding thoracic vertebrae |
| C1980 | Not stated | European shorthair | M | 3 | Diffusely spread hepatic steatosis with periportal necrosis |
| C1990 | Not stated | Not stated | M, n | 8 | Adenocarcinoma in the stomach; old fracture in the distal part of the right ulna with neoartrosis; muscle atrophy right jaw and right front leg |
| C2000 | Vattholma | Domestic shorthair | M, n | 18 | Discospondylitis |
| C2010 | Uppsala | Domestic shorthair | M | 15 | Meningioma; adenoma originating from the pancreatic islets |

M: male; n: neutered.
