# Supplementary material for: Evidence of rustrela virus-associated feline staggering disease in Sweden since the 1970s
Source: Acta Vet Scand. 2024 Nov 23;66:59. doi: 10.1186/s13028-024-00783-5 (PMC11585236; doi:10.1186/s13028-024-00783-5)
Supplement: Supplementary file 4 — Additional file 4: Detection of rustrela virus (RusV) in formalin-fixed, paraffin-embedded brain and spinal cord from cats with non-suppurative meningoencephalitis, controls and reference cases File format: Microsoft Word. File extension. [file 13028_2024_783_MOESM4_ESM.docx]

**Additional file 4.** Detection of rustrela virus (RusV) in formalin-fixed, paraffin-embedded brain and spinal cord from cats with non-suppurative meningoencephalitis, controls and reference cases

|  | **Tissue** | **RusV detection method** | | | | |  |
| --- | --- | --- | --- | --- | --- | --- | --- |
|  |  | **RT-qPCR**  **(Cq value)** | **Immunohistochemistry**  **(grade)** | | | |  |
| **Case No., non-suppurative**  **meningoencephalomyelitis** | | | | | | |  |
| **1978a** | cerebrum | 34.16 | 3 | | | |  |
|  | brain stem | neg. | 2 | | | |  |
|  | cerebellum | 37.01 | 2 | | | |  |
|  | spinal cord | n/a | 2 | | | |  |
| **1978b** | cerebrum | 34.55 | 3 | | | |  |
|  | cerebellum | 37.30 | 2 | | | |  |
|  | cerebellum  and brain stem | 34.81 | cerebellum: 1 | | brain stem: 3 | |  |
|  | spinal cord | n/a | 2 | | | |  |
| **1980** | cerebrum | 35.39 | 0 | | | |  |
|  | cerebellum and brain stem | 36.75 | cerebellum: 2 | | brain stem: 2 | |  |
|  | spinal cord | n/a | 0 | | | |  |
| **1983** | cerebrum | 35.05 | 2 | | | |  |
|  | cerebellum and brain stem | 34.93 | cerebellum: 3 | | brain stem: 3 | |  |
|  | spinal cord | n/a | 2 | | | |  |
| **1984** | cerebrum | 26.08 | 3 | | | |  |
|  | spinal cord | n/a | 3 | | | |  |
| **1990** | brain stem | neg. | 1 | | | |  |
|  | cerebrum, cerebellum and brain stem | neg. | cerebellum: 3 | cerebrum:  0 | | brain stem: 0 | |
|  | cerebrum | neg. | 0 | | | |  |
|  | spinal cord | n/a | 0 | | | |  |
| **1993** | cerebrum | 34.56 | 1 | | | |  |
|  | cerebellum and brain stem | neg. | cerebellum: 3 | | brain stem: 1 | |  |
|  | brain stem | neg. | 1 | | | |  |
|  | spinal cord | n/a | 0 | | | |  |
| **1996** | cerebrum | 33.08 | 3 | | | |  |
|  | cerebrum | 32.55 | 3 | | | |  |
|  | cerebellum and brain stem | 36.51 | cerebellum: 3 | | brain stem: 3 | |  |
|  | spinal cord | n/a | 3 | | | |  |
| **2003** | cerebrum | 29.30 | 3 | | | |  |
|  | cerebrum | 31.81 | 3 | | | |  |
|  | cerebellum and brain stem | 31.43 | cerebellum: 2 | | brain stem: 2 | |  |
|  | spinal cord | n/a | 3 | | | |  |
| **2004** | cerebrum | 33.02 | 1 | | | |  |
|  | cerebellum and brain stem | 33.20 | cerebellum: 1 | | brain stem: 2 | |  |
|  | brain stem | neg. | 1 | | | |  |
|  | spinal cord | n/a | 0 | | | |  |
| **2009** | cerebrum and spinal cord | 34.74 | 0 | | | |  |
|  | cerebrum | 34.95 | 0 | | | |  |
|  | cerebellum, brain stem and spinal cord | 36.80 | cerebellum: 1 | brain stem: 1 | | spinal cord: 1 | |
| **2014** | cerebellum and brain stem | neg. | 0 | | | |  |
|  | cerebrum | neg. | 0 | | | |  |
|  | spinal cord | n/a | 0 | | | |  |
| **2015** | cerebrum | 35.84 | 2 | | | |  |
|  | cerebrum and brain stem | 33.04 | 3 | | | |  |
|  | cerebellum and brain stem | 34.12 | 3 | | | |  |
|  | spinal cord | n/a | 3 | | | |  |
| **2016** | cerebrum | 25.41 | 3 | | | |  |
|  | cerebellum and brain stem | 28.62 | cerebellum: 3 | | brain stem: 3 | |  |
|  | spinal cord | n/a | 3 | | | |  |
| **Case No., controls** | | | | | | |  |
| **C1977** | cerebrum and spinal cord | neg. | 0 | | | |  |
|  | cerebellum | neg. | 0 | | | |  |
|  | brain stem | neg. | 0 | | | |  |
| **C1980** | cerebrum, cerebellum and brain stem | neg. | 0 | | | |  |
| **C1990** | cerebellum and brain stem | neg. | 0 | | | |  |
|  | cerebrum | neg. | 0 | | | |  |
| **C2000** | cerebrum | neg. | 0 | | | |  |
|  | cerebellum | neg. | 0 | | | |  |
|  | brain stem | neg. | 0 | | | |  |
|  | spinal cord | n/a | 0 | | | |  |
| **C2010** | cerebrum, cerebellum and brain stem | neg. | cerebrum:  0 | cerebellum: 0 | | brain stem: 0 | |
| **Reference cases***^a^* **used**  **for immunohistochemistry** | | | | | | |  |
| **RusV-positive cat** | spinal cord | 33.34 | 3 | | | |  |
| **RusV-negative cat** | cerebrum | neg. | 0 | | | |  |

*^a^*Matiasek *et al*., Nat Commun. 2023; 14(1):624, n/a: not applicable (not evaluated), neg.: negative.
