# Supplementary material for: Evidence of rustrela virus-associated feline staggering disease in Sweden since the 1970s
Source: Acta Vet Scand. 2024 Nov 23;66:59. doi: 10.1186/s13028-024-00783-5 (PMC11585236; doi:10.1186/s13028-024-00783-5)
Supplement: Supplementary file 5 — Additional file 5: Immunolabeling for rustrela virus (RusV) in extraneural tissue from four known RusV-positive catsa. File format: Microsoft Word. File extension. [file 13028_2024_783_MOESM5_ESM.docx]

**Additional file 5.** Immunolabeling for rustrela virus (RusV) in extraneural tissue from four known RusV-positive cats*^a^*

|  | Tissue | | | | | | | | |
| --- | --- | --- | --- | --- | --- | --- | --- | --- | --- |
| Case No. | Lung | Liver | Spleen | Kidney | Stomach | Small intestine | Large intestine | Pancreas | Lymph node |
| 2017a | neg. | neg. | n/a | neg. | n/a | pos. | n/a | neg. | n/a |
| 2017b | neg. | neg. | n/a | neg. | neg. | neg. | n/a | neg. | neg. |
| 2017c | neg. | neg. | neg. | neg. | neg. | n/a | neg. | n/a | n/a |
| 2019 | neg. | neg. | neg. | neg. | n/a | n/a | n/a | n/a | n/a |

*^a^*Matiasek *et al*., Nat Commun. 2023; 14(1):624, n/a: not applicable (missing sample), neg.: negative, pos.: positive.
